# Psychometric properties of the Japanese version of the Demoralization Scale‐II (DS‐II) in cancer patients

**DOI:** 10.1002/pcn5.70232

**Published:** 2025-10-26

**Authors:** Yu Tamada, Susumu Ohmae, Masaaki Sasaki, Kohei Echizen, Reiko Yasui, Yumiko Hayashida, Satoko Fukushima, Hiroko Shibuya, Jiro Masuya

**Affiliations:** ^1^ Department of Psychiatry Tokyo Medical University Shinjuku‐ku Tokyo Japan; ^2^ Department of Psychiatry Toranomon Hospital Minato‐ku Tokyo Japan; ^3^ Department of Psychiatry Toranomon Hospital Kajigaya Kawasaki Kanagawa Japan; ^4^ Department of Nursing Toranomon Hospital Minato‐ku Tokyo Japan; ^5^ Department of Nursing Tokyo Medical University Hachioji Medical Center Hachioji Tokyo Japan; ^6^ Cancer Counseling and Support Center, Tokyo Medical University Hachioji Medical Center Hachioji Tokyo Japan

**Keywords:** cancer, demoralization, depression, palliative care, validation

## Abstract

**Aim:**

Demoralization, which is characterized by helplessness, hopelessness, and the loss of meaning, has gained increasing attention in psychiatry and palliative care. Robinson et al. developed the Demoralization Scale‐II (DS‐II) to assess this construct in a brief and reliable format. However, a validated Japanese version has not been developed to date. The purpose of this study was to develop a Japanese version of the DS‐II (DS‐II‐J) and to evaluate its psychometric properties in cancer patients.

**Methods:**

A total of 147 cancer patients from 3 acute care hospitals in Japan were included in the study. Participants completed the DS‐II‐J, Patient Health Questionnaire‐9 (PHQ‐9), Generalized Anxiety Disorder 7‐item Scale (GAD‐7), and Edmonton Symptom Assessment System Revised Version (ESAS‐r). Internal consistency was assessed using Cronbach's *α*. Convergent validity was evaluated by examining correlations with the PHQ‐9, GAD‐7, and ESAS‐r. Confirmatory factor analysis (CFA) and exploratory factor analysis (EFA) were conducted to assess factor structure.

**Results:**

The DS‐II‐J demonstrated high internal consistency (*α* = 0.92). Strong positive correlations were found between DS‐II‐J scores and PHQ‐9 and GAD‐7 scores, and moderate correlations with selected ESAS‐r physical symptoms, supporting convergent validity. CFA results showed a suboptimal model fit for both a one‐factor model and the original two‐factor model proposed by Robinson et al. EFA supported a two‐factor structure, but with a different item composition, suggesting cultural influences.

**Conclusion:**

The DS‐II‐J is a valid and reliable tool for assessing demoralization in Japanese cancer patients.

## INTRODUCTION

In recent years, the concept of demoralization has garnered increasing attention in the fields of psychiatry and palliative care. According to Frank,^1^, ^2^ who first conceptualized demoralization in the context of psychiatry, it is a psychological state characterized by feelings of helplessness, hopelessness, a sense of isolation, and diminished self‐esteem, arising from repeated failures in coping with stress. This condition is generally considered to be more amenable to psychotherapeutic interventions than to pharmacological treatments.^3^ According to the DSM‐5‐TR,^4^ demoralization is often diagnosed as major depressive disorder or adjustment disorder. However, recognizing the features of demoralization may help guide the selection of more appropriate treatment strategies.^5^ Moreover, demoralization has been shown to be closely associated with suicide, making it a potentially useful concept for the early identification of suicide risk.^6^

To enable the empirical assessment of demoralization, Kissane et al.^7^ developed the Demoralization Scale (DS) in 2004. This 24‐item self‐report questionnaire has been translated into multiple languages and is now widely used internationally. In 2016, Robinson et al.^8^ further refined the original DS to reduce patient burden, and developed the Demoralization Scale‐II (DS‐II), which is a 16‐item self‐report questionnaire. The DS‐II comprises two subscales, namely, “meaning and purpose” and “distress and coping ability.” Each item is rated on a three‐point scale ranging from 0 (never) to 2 (often). The DS‐II has also been translated into numerous languages, including Spanish,^9^, ^10^ German,^11^, ^12^ Chinese,^13^, ^14^ Korean,^15^ Greek,^16^ and Persian.^17^, ^18^, ^19^

Regarding the convergent validity of the DS‐II, it has been shown to be positively correlated with depression and anxiety, with correlation coefficients typically ranging from 0.65 to 0.72.^9^, ^11^, ^19^ The internal consistency of the 16‐item DS‐II, as measured by Cronbach's *α*, has been reported to be high, ranging from 0.81 to 0.94.^8^, ^9^, ^10^, ^11^, ^12^, ^14^, ^15^, ^17^, ^19^ Regarding the factor structure of the DS‐II, although several studies^14^, ^17^, ^18^ have supported the original two‐factor structure proposed by Robinson et al.^8^ through confirmatory factor analysis (CFA), other studies have reported different models, including a one‐factor structure,^11^, ^12^, ^19^ a three‐factor structure,^10^ or a two‐factor structure in which the item composition differed from that of Robinson et al.^16^

In recent years, demoralization has gained attention as a characteristic psychological state among cancer patients. In Japan, cancer is one of the most clinically important illnesses, remaining the leading cause of death for over 40 years. Approximately 40% of cancer patients experience depressive conditions, such as major depressive disorder or adjustment disorder, and these depressive states are considered the most important risk factor for suicide among cancer patients.^20^ It is presumed that some of these depressive states may, in fact, reflect demoralization. Despite the importance of recognizing demoralization in Japan, no empirically validated instrument for its assessment exists at present. Therefore, the aim of the present study was to develop a Japanese version of the DS‐II (DS‐II‐J), and to investigate its psychometric properties in a sample of cancer patients.

## METHODS

### Subjects

Participants were consecutively recruited from the following three acute care general hospitals in Japan: Toranomon Hospital, Toranomon Hospital Kajigaya, and Tokyo Medical University Hachioji Medical Center. At Toranomon Hospital and Toranomon Hospital Kajigaya, patients with hematologic malignancies who were admitted for hematopoietic stem cell transplantation between September 2023 and October 2024 were included. In these two hospitals, psychiatric screening is routinely conducted on patients with hematologic malignancies by psychiatrists before transplantation, and patients eligible to enroll were invited to participate in the study during the screening. At Tokyo Medical University Hachioji Medical Center, participants were cancer patients who had been referred for psychiatric consultation between April and August 2023.

The inclusion criteria of the patients were as follows: (1) 18 years old or older, (2) sufficient capacity to consent, and (3) adequate comprehension of Japanese. The exclusion criteria were as follows: (1) severe physical symptoms, (2) impaired consciousness (e.g., delirium), (3) cognitive impairment, (4) severe psychiatric symptoms, and (5) severe suicidal ideation.

The required sample size for validity testing was calculated using G*Power 3.1 software. For a correlation analysis assuming a medium effect size (*r* = 0.30), power of 0.80, and *α* of 0.05, a sample of 82 participants was required. Regarding factor analysis, Hair et al.^21^ recommend a minimum of 100 participants and 5–10 cases per variable. Accordingly, 80–160 participants were considered necessary, and the target sample size was set at 150.

For all participants, data were collected on age, sex, marital status, employment status, living alone (yes/no), number of offspring, years of education, comorbid psychiatric disorders, and family history of psychiatric illness. Cancer‐associated variables included cancer type, initial versus recurrent diagnosis, and time since diagnosis. Psychiatric status was assessed by psychiatrists with more than 10 years of clinical experience based on the DSM‐5, and physical functioning was evaluated using Karnofsky performance status.

Written informed consent was obtained from all participants. The study was conducted in accordance with the Declaration of Helsinki and approved by the Ethics Committee of Tokyo Medical University (approval number: T2023‐0001).

### Assessment measures

All participants completed the following four self‐administered questionnaires.

#### Demoralization Scale‐II

**Figure 1:**
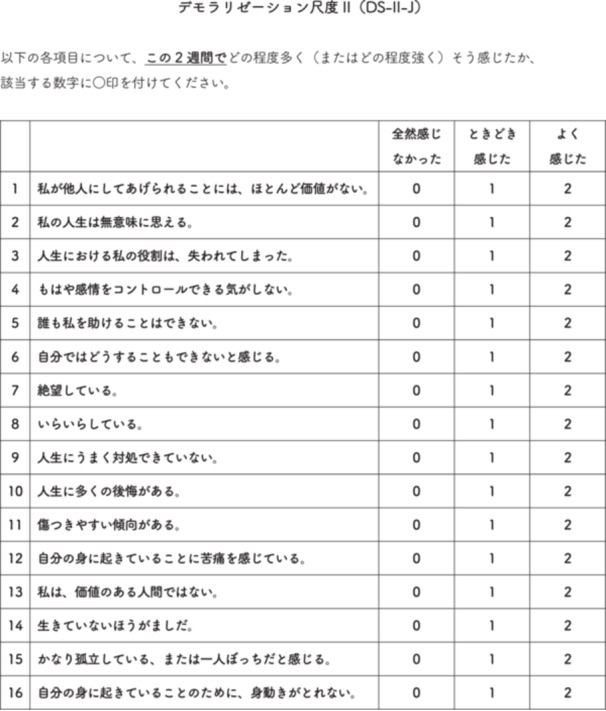
The Japanese version of the Demoralization Scale‐II (DS‐II‐J).

The authors obtained permission from Professor Kissane to translate the DS‐II into Japanese. The translation followed the guidelines provided by the International Society for Pharmacoeconomics and Outcomes Research (ISPOR).^22^ Two psychiatrists fluent in both Japanese and English translated the scale from English to Japanese. The research team reviewed and adjusted the terminology, after which a professional translator performed a back‐translation into English. Professor Kissane reviewed the back‐translated version, and further adjustments were made as necessary. Cognitive debriefing was conducted with five cancer patients at Tokyo Medical University Hachioji Medical Center to ensure clarity and comprehension of the items. Based on these processes, the DS‐II‐J was finalized (Figure 1).

#### Patient Health Questionnaire‐9

The Patient Health Questionnaire‐9 (PHQ‐9), developed by Spitzer et al.,^23^ is a self‐administered scale designed to screen for major depressive disorder in primary care settings. It also assesses the severity of depressive symptoms.^24^ The scale consists of nine items, each rated on a 0–3 scale, yielding a total score ranging from 0 to 27. In this study, the Japanese version of the PHQ‐9^25^ was used, and the total score was utilized to assess the severity of depressive symptoms.

#### Generalized Anxiety Disorder 7‐item Scale

The Generalized Anxiety Disorder 7‐item Scale (GAD‐7), developed by Spitzer et al.,^26^ is a self‐administered questionnaire designed to screen for generalized anxiety disorder and assess the severity of anxiety symptoms. It comprises seven items, each rated on a 0–3 scale, with total scores ranging from 0 to 21. In this study, the Japanese version of the GAD‐7^27^ was used, and the severity of anxiety symptoms was determined from the total score.

#### Edmonton Symptom Assessment System Revised Version

The Edmonton Symptom Assessment System (ESAS), originally developed by Bruera et al.,^28^ is a self‐administered scale for assessing symptoms in cancer patients. The revised version, Edmonton Symptom Assessment System Revised Version (ESAS‐r),^29^ includes the following nine symptoms: pain, tiredness, nausea, depression, anxiety, drowsiness, appetite, well‐being, and shortness of breath, each rated on an 11‐point scale from 0 to 10. There is also an additional item that enables patients to report other symptoms. In this study, the Japanese version of the ESAS‐r^30^ was used, excluding the items for depression, anxiety, and other problems, to assess physical symptoms.

### Data analysis

First, the associations between demographic/clinical variables and the total DS‐II‐J score were investigated using Spearman's rank correlation and two‐sample *t*‐tests. Second, internal consistency of the DS‐II‐J was assessed using Cronbach's *α*. Third, convergent validity was evaluated by calculating Spearman's correlations between DS‐II‐J total scores and scores on the PHQ‐9, GAD‐7, and ESAS‐r. Finally, both CFA and exploratory factor analysis (EFA) were conducted to investigate the factor structure of the DS‐II‐J. CFA was performed using Mplus version 8.5 software (Muthén & Muthén, Los Angeles, CA, USA), and all other analyses were conducted using spss Statistics version 28 software (IBM, Armonk, NY, USA). Maximum likelihood estimation was used for CFA. Model fit was evaluated using the chi‐square test, root mean square error of approximation (RMSEA), comparative fit index (CFI), Tucker–Lewis index (TLI), and standardized root mean square residual (SRMR). A good model fit was defined as a CFI of 0.90 or greater, TLI of 0.90 or greater, RMSEA of 0.08 or less, and SRMR of 0.08 or lessn's^31^ EFA was also conducted using maximum likelihood estimation. Direct oblimin rotation was applied to allow for correlations among factors. The number of factors was determined based on the Kaiser–Guttman criterion and scree plot analysis. The Kaiser–Meyer–Olkin (KMO) measure and Bartlett's test of sphericity were used to assess the suitability of the data for factor analysis. A KMO value of 0.50 or greater was considered acceptable.^32^ A *p*‐value of less than 0.05 was considered to indicate a statistically significant difference between groups.

## RESULTS

### Demographic and clinical characteristics, and DS‐II‐J total scores of the patients

A total of 153 patients agreed to participate in the study and completed the self‐administered questionnaires. However, six patients with missing data on the DS‐II‐J were excluded, resulting in 147 participants being included in the analysis. Demographic and clinical characteristics, together with DS‐II‐J scores, are presented in Table 1. A significant negative correlation was observed between the number of offspring and DS‐II‐J total scores. We also compared patients with hematological malignancies (Toranomon Hospital and Toranomon Hospital Kajigaya) and those referred for psychiatric consultation (Tokyo Medical University Hachioji Medical Center). Mean PHQ‐9 scores were 5.6 (SD = 4.6) and 5.8 (SD = 4.7), and mean DS‐II‐J scores were 6.3 (SD = 6.1) and 5.9 (SD = 7.6), respectively. No significant differences were observed between the two groups.

**Table 1:** Demographic and clinical characteristics and Japanese version of the Demoralization Scale‐II (DS‐II‐J) scores of the 147 cancer patients in this study.

Characteristic or measure	Value (number or mean ± SD)	Correlation with total DS‐II‐J score (*ρ*)	Effect on total DS‐II‐J score (mean ± SD, *t*‐test)	*p* value
Age, years	57.1 ± 13.1	*ρ* = −0.16	—	0.06
Sex (male:female)	89:58	—	Male (5.9 ± 6.0) versus female (6.7 ± 6.6)	0.45
Education, years	14.6 ± 2.1	*ρ* = 0.05	—	0.56
Employment status (employed:unemployed)	75: 72	—	Employed (6.2 ± 6.2) versus unemployed (6.3 ± 6.3)	0.88
Marital status (married:unmarried)	110:37	—	Married (5.7 ± 5.7) versus unmarried (7.9 ± 7.3)	0.06
Living alone (yes:no)	23:124	—	Yes (7.9 ± 7.1) versus no (5.9 ± 6.0)	0.17
Number of offspring	1.4 ± 1.1	*ρ* = −0.19^a^	—	0.02
Comorbid psychiatric disorder (yes:no)	27:120	—	Yes (8.3 ± 7.8) versus no (5.8 ± 5.7)	0.13
First‐degree relative with psychiatric disorder (yes:no)	21:126	—	Yes (7.3 ± 6.8) versus no (6.1 ± 6.1)	0.40
Tumor site				
Hematological	137	—	—	—
Lung	4	—	—	—
Breast	2	—	—	—
Gastric	1	—	—	—
Rectal	1	—	—	—
Pharyngeal	1	—	—	—
Prostate	1	—	—	—
Disease phase			—	—
Primary cancer	89	—	—	—
Recurrence	51	—	—	—
Multiple primary cancers	7	—	—	—
Months since first cancer diagnosis	21.9 ± 33.2	*ρ* = 0.01	—	0.92
Months since current cancer/cancer recurrence diagnosis	9.6 ± 25.4	*ρ* = 0.05	—	0.54
Karnofsky performance scale	73.7 ± 15.0	*ρ* = −0.09	—	0.27

*Note*: Data are presented as means ± SD or numbers. *ρ* = Spearman's correlation coefficient.

Abbreviation: SD, standard deviation.

^a^ *p* < 0.05.

### Internal consistency of the DS‐II‐J

Cronbach's *α* for the DS‐II‐J total scale was 0.92. For the subscales proposed by Robinson et al., Cronbach's *α* was 0.87 for “meaning and purpose,” and 0.86 for “distress and coping ability.” The correlation between the two subscales was *ρ* = 0.73 (*p* < 0.001).

### Convergent validity of the DS‐II‐J with other measures

Total scores on the DS‐II‐J, its original subscales, and the PHQ‐9, GAD‐7, and ESAS‐r are presented in Table 2. The DS‐II‐J total score showed very strong positive correlations with both PHQ‐9 and GAD‐7 scores. Regarding correlations between the DS‐II‐J total score and individual items of the ESAS‐r, a strong positive correlation was observed with well‐being, moderate positive correlations with tiredness, lack of appetite, nausea, and shortness of breath, and weak positive correlations with pain and drowsiness.

**Table 2:** Scores of the self‐reported questionnaires in the 147 cancer patients.

Measure	Value (mean ± SD)	Correlation with total DS‐II‐J score
DS‐II‐J total score	6.2 ± 6.2	—
Meaning and purpose subscale score	2.7 ± 3.1	—
Distress and coping ability subscale score	3.6 ± 3.5	—
PHQ‐9 total score	5.6 ± 4.6	*ρ* = 0.71^a^
GAD‐7 total score	3.4 ± 3.6	*ρ* = 0.68^a^
ESAS‐r scores		
Pain	1.5 ± 2.2	*ρ* = 0.34^a^
Tiredness	2.2 ± 2.3	*ρ* = 0.58^a^
Drowsiness	1.9 ± 2.0	*ρ* = 0.34^a^
Nausea	0.7 ± 1.8	*ρ* = 0.41^a^
Lack of appetite	1.8 ± 2.6	*ρ* = 0.45^a^
Shortness of breath	0.8 ± 1.7	*ρ* = 0.41^a^
Well‐being	3.4 ± 2.4	*ρ* = 0.61^a^

*Note*: Data are presented as means ± SD. *ρ* = Spearman's correlation coefficient.

Abbreviations: DS‐II‐J, Japanese version of the Demoralization Scale‐II; ESAS‐r, Edmonton Symptom Assessment System‐revised; GAD‐7, Generalized Anxiety Disorder 7‐item scale; PHQ‐9, Patient Health Questionnaire‐9, SD, standard deviation.

^a^ *p* < 0.01.

### Factor analysis

CFA was conducted to analyze the factor structure of the 16‐item DS‐II‐J, testing both a one‐factor model and the original two‐factor model proposed by Robinson et al. The results are presented in Table 3. The fit indices for the one‐factor model were not satisfactory. The original two‐factor model showed a slightly better fit than the one‐factor model, but the overall fit was still insufficient. Next, EFA was conducted to investigate the factor structure of the DS‐II‐J. The number of factors was determined using the Kaiser–Guttman criterion (eigenvalues > 1) and scree plot, both of which supported a two‐factor structure. The correlation between the two factors was 0.68, indicating a moderate to strong association. Detailed factor loadings are shown in Table 4. Items with loadings 0.40 and greater were considered to load significantly on the corresponding factor. Although Item 5 had a loading slightly below 0.40, it was judged to belong to Factor 1. Item 9 showed cross‐loadings on both factors (≥0.40) and was assigned to Factor 2, in which the loading was higher. The KMO measure indicated excellent sampling adequacy (KMO = 0.916), and Bartlett's test of sphericity was significant (χ² = 1287.7, *p* < 0.001).

**Table 3:** Fit indices for the one‐factor model and the original two‐factor model.

	*χ*² (df)	CFI	TLI	RMSEA (90% CI)	SRMR
One‐factor model	293.3 (104)	0.847	0.823	0.111 (0.096–0.126)	0.066
Two‐factor model	259.8 (103)	0.873	0.852	0.102 (0.086–0.117)	0.066

Abbreviations: *χ*², chi‐square test; CFI, comparative fit index; CI, confidence interval; df, degrees of freedom; RMSEA, root mean square error of approximation; SRMR, standardized root mean squared residual; TLI, Tucker–Lewis index.

**Table 4:** Pattern matrix from the exploratory factor analysis of the Japanese version of the Demoralization Scale‐II (DS‐II‐J).

Item		Factor 1	Factor 2
12	I feel distressed about what is happening to me.	0.831	0.031
6	I feel that I cannot help myself.	0.715	0.873
11	I tend to feel hurt easily.	0.643	0.067
16	I feel trapped by what is happening to me.	0.624	0.108
10	I have a lot of regret about my life.	0.505	0.173
8	I feel irritable.	0.471	0.022
4	I no longer feel emotionally in control.	0.465	0.197
7	I feel hopeless.	0.428	0.344
5	No one can help me.	0.388	0.334
2	My life seems to be pointless.	0.175	0.970
13	I am not a worthwhile person.	0.014	0.860
14	I would rather not be alive.	0.054	0.780
3	My role in life has been lost.	0.115	0.603
15	I feel quite isolated or alone.	0.309	0.509
1	There is little value in what I can offer others.	0.145	0.474
9	I do not cope well with life.	0.418	0.445

*Note*: Extraction method: maximum likelihood; Rotation: oblimin with Kaiser normalization.

## DISCUSSION

In this study, the first DS‐II‐J was developed through a rigorous process of translation and validation, and its psychometric properties were evaluated. Among the demographic variables, a negative correlation was found between DS‐II‐J scores and the number of offspring. Contrary to a previous study,^33^ no significant associations were observed between DS‐II‐J scores and employment status, marital status, cohabitation, or years of education. However, DS‐II‐J scores tended to be higher among unmarried participants than married participants (*p* = 0.06). Although this difference was not statistically significant, it may suggest that unmarried individuals are more likely to require psychological support in clinical settings.

In addition, although recruitment settings differed across hospitals, PHQ‐9 and DS‐II‐J scores did not significantly differ between patients with hematological malignancies and those referred for psychiatric consultation. This suggests that levels of depression and demoralization were comparable across these populations, although the small sample size warrants cautious interpretation.

The DS‐II‐J total score showed very strong correlations with the severity of depression and anxiety, supporting its convergent validity. Consistent lines of evidence suggests that demoralization is associated with physical problems,^33^ and in the present study, positive correlations between DS‐II‐J scores and ESAS‐r items further support its convergent validity. In addition, the DS‐II‐J demonstrated high internal consistency for both the total scale and the two subscales proposed by Robinson et al.

With regard to the factor structure, the results of the CFA raised questions about whether the original two‐factor model proposed by Robinson et al. could be directly applied to the DS‐II‐J. Although the EFA supported a two‐factor solution, the composition of items differed from the original structure. Regarding Factor 2 in the present study, Items 5, 6, and 7 were excluded from the “meaning and purpose” subscale, whereas Items 9 and 15 were included. This suggests that, in the Japanese version, “loss of meaning and purpose” and “feelings of isolation” may have been integrated into a single factor (Factor 2).

On the other hand, Items 5, 6, and 7, which loaded on Factor 1, primarily reflect helplessness and hopelessness. This suggests that, in the DS‐II‐J, these elements are more strongly associated with “distress” than in the original DS‐II. Regarding coping ability, Item 4 was classified under Factor 1, whereas Item 9 cross‐loaded on both factors with loadings above 0.40. These findings indicate that Factor 1 may represent a construct different from that proposed by Robinson et al., suggesting the need to reconsider its label.

Previous studies on the factor structure of the DS‐II have yielded inconsistent results.^10^, ^11^, ^16^, ^19^ This suggests that the subscale structure of the DS‐II may vary depending on cultural factors. In particular, the classification of “loss of meaning and purpose” and “feelings of isolation” under the same factor may reflect a cultural linkage between loss of meaning and social isolation in Japanese society.

In the Japanese cultural context, loss of meaning and purpose and feelings of isolation may be more closely interconnected than in Western settings, which may explain why these items clustered together in Factor 2 of the DS‐II‐J. Similar discrepancies in factor structure have been reported in other language versions, such as a one‐factor model in Germann's^11^, ^12^ a three‐factor model in Spanish,^10^ and modified two‐factor models in Greek version,^16^ suggesting that cultural and linguistic contexts shape the conceptualization of demoralization. In our EFA, Factor 1 primarily captured helplessness, hopelessness, and emotional distress, whereas Factor 2 reflected loss of meaning and isolation. These results indicate that Factor 1 could be interpreted as “distress and hopelessness,” and Factor 2 as “loss of meaning and isolation,” underscoring culturally specific features of demoralization in the Japanese context.

In addition, the two‐factor model proposed by Robinson et al.^8^ was derived from patients with progressive disease or in palliative care. In contrast, our sample consisted mainly of patients with hematological malignancies undergoing hematopoietic stem cell transplantation and cancer patients referred for psychiatric consultation, who were not necessarily in a palliative care setting. In palliative care, demoralization and meaning‐making are often shaped by existential concerns such as facing death and reevaluating life's meaning, whereas in transplantation or consultation settings, treatment‐related anxiety and isolation may play a more prominent role. Therefore, the differences in factor structure observed in our study may reflect not only cultural influences but also differences in clinical background. Further investigation in future research is warranted to clarify this point.

This study has several limitations. First, most participants were patients with hematologic malignancies, which may have led to sample bias. The psychological experience of demoralization may differ between patients with hematological malignancies and those with solid tumors. For example, patients undergoing hematopoietic stem cell transplantation are often placed in protective isolation, which can cause unique psychological burdens such as feelings of disconnection and loss of control.^34^, ^35^ In contrast, patients with solid tumors may face different existential concerns. Therefore, caution is warranted when generalizing the present findings, and future validation studies involving more diverse cancer populations are needed. Second, test–retest reliability was not assessed. This decision was made to minimize participant burden and in consideration of the context‐dependent nature of demoralization. Future studies should investigate the clinical characteristics of demoralization not only in cancer patients but also in individuals with other physical or mental illnesses.

In conclusion, the 16‐item DS‐II‐J demonstrated acceptable validity and internal consistency, indicating sound psychometric properties. However, the factor structure of the subscales may vary depending on the version of the scale and the characteristics of the target population, warranting cautious interpretation.

## AUTHOR CONTRIBUTIONS

**Yu Tamada**: Conceptualization; methodology; formal analysis; investigation; data curation; writing—original draft; project administration; funding acquisition. **Susumu Ohmae**: Conceptualization; data curation; writing—review and editing; supervision. **Masaaki Sasaki**: Investigation; data curation; writing—review and editing. **Kohei Echizen**: Investigation; data curation; writing—review and editing. **Reiko Yasui**: Investigation; data curation; writing—review and editing. **Yumiko Hayashida**: Investigation; data curation; writing—review and editing. **Satoko Fukushima**: Investigation; data curation; writing–review and editing. **Hiroko Shibuya**: Investigation; data curation; writing—review and editing. **Jiro Masuya**: Conceptualization; writing–review and editing; supervision. All authors contributed to and have approved the final manuscript.

## CONFLICT OF INTEREST STATEMENT

The authors declare no conflicts of interest.

## ETHICS APPROVAL STATEMENT

This study protocol was reviewed and approved by the Ethics Committee of Tokyo Medical University (approval number: T2023‐0001).

## PATIENT CONSENT STATEMENT

All participants provided written consent to participate in the study.

## CLINICAL TRIAL REGISTRATION

N/A.

## Data Availability

The data that support the findings of this study are available on request from the corresponding author. The data are not publicly available due to privacy or ethical restrictions.

## References

[1] Frank JD . Persuasion and healing: a comparative study of psychotherapy, revised edition. Baltimore: Johns Hopkins University Press; 1973.

[2] Frank JD . Psychotherapy: the restoration of morale. Am J Psychiatry. 1974;131:271–274. 4812687 10.1176/ajp.131.3.271

[3] Klein DF , Gittelman R , Quitkin F , Rifkin A . Diagnosis and drug treatment of psychiatric disorders: adult and children. 2nd ed. Baltimore: Williams & Wilkins; 1980.

[4] American Psychiatric Association . Diagnostic and statistical manual of mental disorders, fifth edition, text revision. Washington (DC): American Psychiatric Association; 2022.

[5] Kissane DW , Grassi L , Fang CK . Adjustment disorder and demoralization. In: Kissane DW , Watson M , Breitbart WS , editors. Psycho‐oncology in palliative and end of life care. New York: Oxford University Press; 2023.

[6] Costanza A , Vasileios C , Ambrosetti J , Shah S , Amerio A , Aguglia A , et al. Demoralization in suicide: a systematic review. J Psychosom Res. 2022;157:110788. 35334350 10.1016/j.jpsychores.2022.110788

[7] Kissane DW , Wein S , Love A , Lee XQ , Kee PL , Clarke DM . The demoralization scale: a report of its development and preliminary validation. J Palliat Care. 2004;20:269–276. 15690829

[8] Robinson S , Kissane DW , Brooker J , Michael N , Fischer J , Franco M , et al. Refinement and revalidation of the demoralization scale: the DS‐II‐internal validity. Cancer. 2016;122:2251–2259. 27171617 10.1002/cncr.30015

[9] Belar A , Arantzamendi M , Rodríguez‐Núñez A , Santesteban Y , Martinez M , López‐Saca M , et al. Multicenter study of the psychometric properties of the new Demoralization Scale (DS‐II) in Spanish‐speaking advanced cancer patients. J Pain Symptom Manage. 2019;57:627–634. 30472315 10.1016/j.jpainsymman.2018.11.016

[10] Palacios‐Espinosa X , Sánchez Pedraza R , Rodríguez C . Psychometric properties of Demoralization Scale (DS‐II Spanish version‐Colombia) for oncologic patients in palliative care. Av Psicol Latinoam. 2020;38:1–18.

[11] Koranyi S , Hinz A , Hufeld JM , Hartung TJ , Quintero Garzón L , Fendel U , et al. Psychometric evaluation of the German version of the Demoralization Scale‐II and the association between demoralization, sociodemographic, disease‐ and treatment‐related factors in patients with cancer. Front Psychol. 2021;12:789793. 34899543 10.3389/fpsyg.2021.789793 PMC8652041

[12] Ramm M , Jedamzik J , Lenz P , Poopana A , Heuft G , Conrad R . Psychometric properties and normative values of the revised Demoralization Scale (DS‐II) in a representative sample of the German general population. BMC Psychiatry. 2023;23:685. 37730585 10.1186/s12888-023-05187-9 PMC10512641

[13] Na OU , Xiaoping HU , Sanyang QI , Xiaoyan LUO , Wenjie NIE . The Chinese version of the Demoralization Scale‐II: development, reliability and validity in Chinese cancer patients. Chin Gen Pract. 2021;24:2998–3004.

[14] Wu WJ , Quan M , Gao L , Li Q , Yan CX , Zhang Q , et al. Demoralization and depression in Chinese cancer patients. Support Care Cancer. 2021;29:6211–6216. 33834301 10.1007/s00520-021-06195-9

[15] Jhon M , Kim SY , Kim JM , Kim SW , Shin IS , Lee JY , et al. Standardization and validation of the Korean version of Demoralization Scale‐II (DS‐II‐Kr) in cancer patients. J Korean Neuropsychiatr Assoc. 2022;61:90–97.

[16] Elmasian TF , Nikoloudi M , Tsilika E , Kostopoulou S , Zygogianni A , Katsaragakis S , et al. Psychometric properties of the Greek version of Demoralization Scale‐II (DS‐II) in patients with cancer. J Caring Sci. 2023;12:103–109. 37469748 10.34172/jcs.2023.31856 PMC10352633

[17] Foroughi A , Khanjani S , Soleymani Moghadam M , Parvizifard A . The psychometric properties of the Persian version of the Demoralization Scale (DS‐II) in women with breast cancer. J Res Med Sci. 2024;29:14. 38808221 10.4103/jrms.jrms_94_23 PMC11132418

[18] Mousavi N , Piryaei M , Nooripour R , Kissane D , Hooshyari Z , Effatpanah M , et al. Psychometric properties of Farsi version of Demoralization Scale‐II (DS‐II) in Iranian cancer patients. Palliat Support Care. 2024;22:1825–1834. 10.1017/S1478951524000129 39620652

[19] Taghilou E , Heidarzadeh M , Molaei B , Khameslou MA . Determining psychometric properties of the Persian version of Demoralization Scale‐II in patients with cancer. BMC Psychol. 2024;12(1):1. 38167530 10.1186/s40359-023-01507-6 PMC10759485

[20] Akechi T . Depression in patients with cancer. Gendai Igaku (Curr Med). 2022;69:30–35.

[21] Hair JF , Black WC , Babin BJ , Anderson RE . Multivariate data analysis: a global perspective. 7th ed. Upper Saddle River: Pearson; 2010.

[22] Wild D , Grove A , Martin M , Eremenco S , McElroy S , Verjee‐Lorenz A , et al. Principles of good practice for the translation and cultural adaptation process for Patient‐Reported Outcomes (PRO) measures: report of the ISPOR Task Force for Translation and Cultural Adaptation. Value Health. 2005;8:94–104. 15804318 10.1111/j.1524-4733.2005.04054.x

[23] Spitzer RL , Kroenke K , Williams JB , the Patient Health Questionnaire Primary Care Study Group . Validation and utility of a self‐report version of PRIME‐MD: the PHQ primary care study. JAMA. 1999;282:1737–1744. 10568646 10.1001/jama.282.18.1737

[24] Kroenke K , Spitzer RL , Williams JBW . The PHQ‐9: validity of a brief depression severity measure. J Gen Intern Med. 2001;16:606–613. 11556941 10.1046/j.1525-1497.2001.016009606.x PMC1495268

[25] Muramatsu K , Muramatsu K , Kamijima K , Yoshida M , Otsubo T , Miyaoka H , et al. The patient health questionnaire, Japanese version: validity according to the mini‐international neuropsychiatric interview‐plus. Psychol Rep. 2007;101:952–960. 18232454 10.2466/pr0.101.3.952-960

[26] Spitzer RL , Kroenke K , Williams JBW , Löwe B . A brief measure for assessing generalized anxiety disorder: the GAD‐7. Arch Intern Med. 2006;166:1092–1097. 16717171 10.1001/archinte.166.10.1092

[27] Muramatsu K , Miyaoka H , Kamijima K , Muramatsu Y , Fuse K . Investigation of the validity and utility of the Japanese version of the GAD‐7. Shinshin‐igaku. 2010;50:166.

[28] Bruera E , Kuehn N , Miller MJ , Selmser P , Macmillan K . The Edmonton Symptom Assessment System (ESAS): a simple method for the assessment of palliative care patients. J Palliat Care. 1991;7:6–9. 1714502

[29] Watanabe SM , Nekolaichuk CL , Beaumont C . The Edmonton Symptom Assessment System, a proposed tool for distress screening in cancer patients: development and refinement. Psycho‐Oncology. 2012;21:977–985. 21671304 10.1002/pon.1996

[30] Yokomichi N , Morita T , Nitto A , Takahashi N , Miyamoto S , Nishie H , et al. Validation of the Japanese version of the Edmonton Symptom Assessment System‐Revised. J Pain Symptom Manage. 2015;50:718–723. 26169339 10.1016/j.jpainsymman.2015.05.014

[31] Hu L , Bentler PM . Cutoff criteria for fit indexes in covariance structure analysis: conventional criteria versus new alternatives. Struct Equ Modeling. 1999;6:1–55.

[32] Kaiser HF . An index of factorial simplicity. Psychometrika. 1974;39:31–36.

[33] Robinson S , Kissane DW , Brooker J , Burney S . A systematic review of the demoralization syndrome in individuals with progressive disease and cancer: a decade of research. J Pain Symptom Manage. 2015;49:595–610. 25131888 10.1016/j.jpainsymman.2014.07.008

[34] Tecchio C , Bonetto C , Bertani M , Cristofalo D , Lasalvia A , Nichele I , et al. Predictors of anxiety and depression in hematopoietic stem cell transplant patients during protective isolation. Psycho‐Oncology. 2013;22:1790–1797. 23132747 10.1002/pon.3215

[35] Lee RS , Halliday LE . The psychological effects of protective isolation on haematological stem cell transplant patients: an integrative, descriptive review. Support Care Cancer. 2025;33:133. 39888407 10.1007/s00520-025-09186-2 PMC11785653

